# Association of Dietary Vegetable and Fruit Consumption with Sarcopenia: A Systematic Review and Meta-Analysis

**DOI:** 10.3390/nu16111707

**Published:** 2024-05-30

**Authors:** Seung-Hee Hong, Yun-Jung Bae

**Affiliations:** 1 Department of Food and Nutritional Science, Shinhan University, Uijeongbu 11644, Gyeonggi, Republic of Korea; hsh@shinhan.ac.kr; 2 Major in Food and Nutrition, Division of Food Science and Biotechnology, Korea National University of Transportation, Jeungpyeong 27909, Chungcheong, Republic of Korea

**Keywords:** sarcopenia, vegetable, fruit, meta-analysis

## Abstract

Previous studies have shown contradictory results regarding the association between vegetable and fruit consumption and the risk of sarcopenia. We aimed to evaluate this association using a meta-analysis, following the Preferred Reporting Items for Systematic Reviews and Meta-Analyses guidelines. We searched PubMed, EMBASE, and the Cochrane Library through July 2023 using related keywords. Pooled odds ratios (ORs) with 95% confidence intervals (CIs) were calculated based on the random-effects model. We included 14 observational studies with 11 cross-sectional and three cohort studies involving 6436 sarcopenias among 33,801 participants. Vegetable and fruit consumption were significantly associated with reduced sarcopenia risk (OR, 0.61; 95% CI, 0.48 to 0.79; *I*^2^ = 59.8%). The association was significant in cross-sectional studies (OR, 0.64; 95% CI, 0.49 to 0.84; *I*^2^ = 56.3%; n = 11) but not in cohort studies (OR, 0.50; 95% CI, 0.22 to 1.11; *I*^2^ = 76.4%; n = 3). Moreover, the association was significant in age ≥60 (OR, 0.64; 95% CI, 0.49 to 0.83; *I*^2^ = 58.0%; n = 10). This meta-analysis suggests that eating vegetables and fruit reduces sarcopenia risk. However, as cohort studies provide a higher level of evidence than case–control studies, further prospective cohort studies should be conducted.

## 1. Introduction

Sarcopenia is a condition characterized by the gradual loss of muscle mass and strength in skeletal muscles, and the risk of sarcopenia increases with age [1]. The prevalence of sarcopenia is 8–36% in adults aged <60 years and 10–27% in those aged ≥60 years, and the prevalence of severe sarcopenia in adults with an average age of 68.5 years is 2–9% [2]. Sarcopenia is closely related to disability in basic physical functions such as falls, functional decline, and fractures [3,4], and to metabolic abnormalities such as diabetes and hypertension [5,6]. It is also considered as a major predictor of all-cause mortality [7]. Therefore, the prevention and management of sarcopenia is crucial as the risk of sarcopenia increases with age, and it is associated with other chronic diseases.

Factors associated with deterioration in muscle quantity and quality include chronic diseases such as diabetes and stroke, which can lead to changes in muscle tissue [8], physical inactivity [9], malnutrition [10], and dietary factors [11,12]. Changes in dietary intake can affect muscle mass and function. Low protein intake [13], low energy intake [14], and deficiencies in nutrients such as vitamin D and antioxidants are closely associated with changes in muscle mass and function.

Recent studies have found an association between vegetable and fruit consumption and sarcopenia [15,16]. Previous studies have shown that vitamin C and phytochemicals in vegetables and fruits are beneficial factors for muscle synthesis and can enhance muscle recovery [17,18]. Studies on the relationship between vegetable and fruit consumption and sarcopenia have repeatedly reported on dietary patterns, including vegetables and fruit, along with the quantity of vegetables and fruit consumed [19,20,21,22]. To date, only one meta-analysis has been conducted on the association between vegetable and fruit consumption and sarcopenia, focusing on the supplementation of antioxidant-rich foods such as vegetables, fruit, nuts, and seeds using a handgrip strength test [23]. Several studies on the association between vegetable and fruit consumption and sarcopenia showed different levels of significance by sex [15], and inconsistencies in the findings related to vegetables and fruit themselves [24], making it difficult to draw a general conclusion about the association between vegetable and fruit consumption and sarcopenia. Therefore, we conducted a systematic literature review and meta-analysis to clarify the association between vegetable and fruit consumption and sarcopenia.

## 2. Methods

### 2.1. Search Strategy

We performed a systematic review and meta-analysis of observational studies regarding sarcopenia. The present review was in accordance with the Preferred Reporting Items for Systematic Reviews and Meta-analyses guidelines [25]. We systematically searched the MEDLINE, EMBASE, and Cochrane Library databases for eligible studies published up to July 2023. We selected MeSH terms for MEDLINE and keywords related to fruit and vegetable consumption and sarcopenia. The complete search strategy was the following in PubMed: (“Sarcopenia”[MeSH Terms] OR (“Sarcopenia”[Text Word] OR “sarcopenias”[Text Word] OR “skeletal muscle mass”[Text Word] OR “low muscle mass”[Text Word] OR “handgrip strength”[Text Word])) AND (“Vegetables”[MeSH Terms] OR “Fruit”[MeSH Terms] OR “Citrus”[MeSH Terms] OR (“vegetable”[Text Word] OR “Vegetables”[Text Word] OR “Fruit”[Text Word] OR “fruits”[Text Word] OR “plant capsule”[Text Word] OR “capsules plant”[Text Word] OR “plant capsules”[Text Word] OR “berries”[Text Word] OR “berry”[Text Word] OR “legume pod”[Text Word] OR “legume pods”[Text Word] OR “pod legume”[Text Word] OR “Citrus”[Text Word] OR “citrus fruits”[Text Word] OR “cruciferae”[Text Word] OR “cruciferous vegetables”[Text Word] OR “mediterranean diet”[Text Word])).

### 2.2. Eligibility Criteria and Study Selection

Two of the authors independently evaluated the eligibility of all studies retrieved from the database and bibliographies. Disagreements between the reviewers during the selection process were resolved by consensus. The inclusion criteria were the following: (1) observational studies (cross-sectional, case–control, and cohort); (2) assessed all three sarcopenia parameters (low muscle mass, muscle strength, and physical performance); (3) contained information on sarcopenia with the corresponding OR and 95% CI; (4) conducted within the general population; and (5) published in English and human studies. The exclusion criteria were the following: (1) assessed only one or two sarcopenia parameters; (2) consisted only of frail individuals; (3) investigated sarcopenic obesity, as obesity increases the risk of sarcopenia; (4) multiple publications of the same data; and (5) clinical trials, reviews, case reports, and animal studies. If there are multiple articles on the same primary study, then the most comprehensive one was included.

### 2.3. Data Extraction

Observational studies that met all the above criteria were included for data extraction. Two independent authors extracted the following data from studies: first author, year of publication, country of included participants, definition of sarcopenia, body composition assessment method, baseline age (years), dietary assessment, definition of vegetable and fruit consumption, categories of exposure (highest versus lowest), odds ratio (OR) with 95% confidence interval (CI), and adjusted variables.

### 2.4. Main and Subgroup Analyses

In the main analysis, we investigated the association between vegetable and fruit consumption and the risk of sarcopenia using adjusted ORs with 95% CIs. The groups with the highest levels of vegetable and fruit consumption were compared with the groups with the lowest levels in each study. We also performed subgroup analyses according to study design, sex, age, dietary patterns, definition criteria for sarcopenia, geographical region of participants, and methodological quality.

### 2.5. Quality Assessment Based on the Risk of Bias

We assessed the risk of bias of the included studies based on the Newcastle–Ottawa Scale (NOS) [26] for cross-sectional and cohort studies. The NOS scores ranged from 0 to 9 and consist of three subscales: selection (up to 3 points), comparability (up to 2 points), and exposure (up to 3 points), with the highest possible score being 9, reflecting the lowest risk of bias. A score of ≤6 points was classified as low quality, and a score of ≥7 points was classified as high quality.

### 2.6. Statistical Analysis

We used the adjusted OR and 95% CI reported in each study, comparing the highest with the lowest (reference) exposures to calculate the pooled OR with 95% CI. The pooled effect was calculated using the inverse variance-weighted mean of the logarithm of the adjusted OR with 95% CI. We evaluated the heterogeneity of results across studies using Higgins *I*^2^, which measures the percentage of total variation across studies [27]. *I*^2^ ranged from 0% (no observed heterogeneity) to 100% (maximum heterogeneity), and an *I*^2^ value of >50% indicated substantial heterogeneity. We used a random-effects model for all analyses using the DerSimonian and Laird method, as individual studies showed high variability in study design and were conducted in different populations [28]. We also examined the studies included in the final analysis for publication bias using Begg’s funnel plot and Egger’s test. If publication bias existed, Begg’s funnel plot was asymmetric, or the *p*-value was <0.05, as determined by Egger’s test. We performed all statistical analyses using the Stata SE version 16 software package (StataCorp, College Station, TX, USA).

## 3. Results

### 3.1. Selection of Relevant Studies

**Figure 1:**
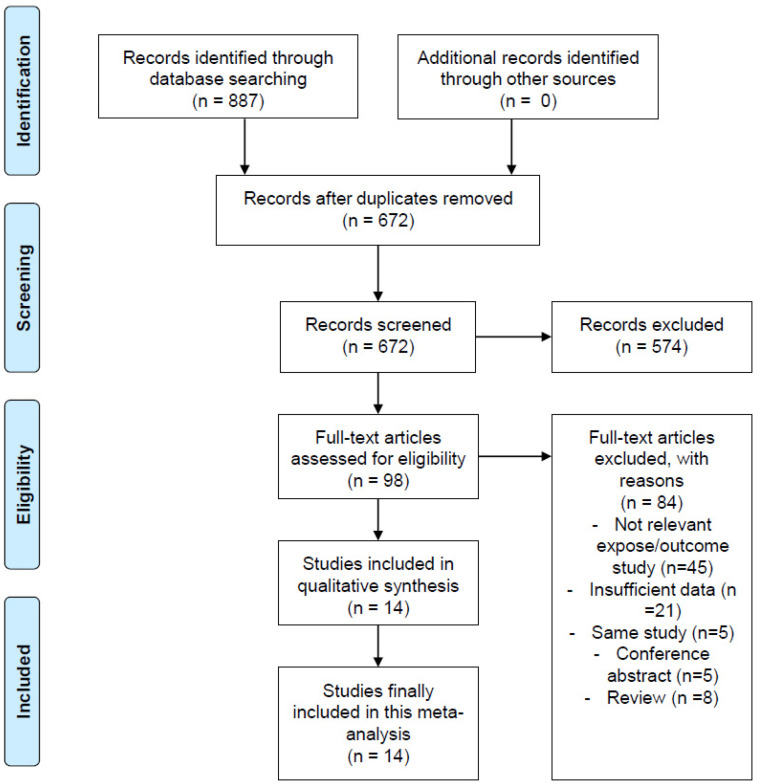
PRISMA 2020 flow chart of the article selection. PRISMA, Preferred Reporting Items for Systematic Reviews and Meta-Analyses.

A total of 887 studies were retrieved from the preliminary database search of all data (Figure 1). As many as 215 studies were excluded due to duplication, and 574 were excluded for not fulfilling the selection criteria during the title and abstract screening. The remaining 98 studies were assessed through a full-text study, and 84 were subsequently excluded for the reasons listed in Figure 1. The remaining 14 studies [15,19,20,24,29,30,31,32,33,34,35,36,37,38] were included in the final meta-analysis.

### 3.2. Characteristics of the Included Studies

Table 1 presents the characteristics of the studies included in the meta-analysis. There were 14 observational studies, which included 6436 patients with sarcopenia among 33,801 participants. The included studies were 11 cross-sectional studies [15,19,20,29,30,31,32,33,35,36,38] and three cohort studies [24,34,37]. Nine studies were conducted with participants aged ≥65 years [15,19,24,31,33,34,35,36,37], and one was conducted with participants aged ≥60 years [38]. For the definition of exposure, three studies were assessed with vegetable and fruit consumption [19,31,34], three with vegetable consumption [15,24,38], and three with the Mediterranean dietary pattern [30,32,36]. Regarding the criteria for defining sarcopenia, the definition from the Asian Working Group for Sarcopenia (AWGS) 2014 was most frequently used (four studies) [24,31,33,38], followed by the definition from the European Working Group on Sarcopenia in Older People 1 (EWGSOP1) (three studies) [29,30,32], EWGSOP2 (three studies) [20,36,37], and AWGS 2019 (two studies) [34,35]. Ten studies were conducted in Asia [19,20,24,30,31,32,33,34,35,38], two in Europe [36,37], and one in America [29].

### 3.3. Methodological Quality

Table 2 shows the individual NOS scores for each criterion in the included studies. The scores of all studies were >5. The mean score was 6.7 for cross-sectional studies and 7.0 for cohort studies. A study was classified as high-quality if it achieved more than the mean score for each study design. Seven studies were categorized as high-quality (score ≥ 7) and seven studies as low-quality (score ≤ 6).

**Table 1:** General characteristics of the studies included in the final analysis.

Study	Country	Definition of Sarcopenia	Body Composition	Participants (Sarcopenia/No Sarcopenia)	Base Line Age (years)	Dietary Assessment	Definition of Vegetables and Fruits Consumption	Categories of Expose (Highest vs. Lowest Category)	OR (95%CI)	Adjusted Variables
Cross-sectional study										
2013 Fanelli [29]	USA	EWGSOP1	DXA	2176 (-/-)	30–64	24 h dietary recall. Interviewer administered	Starchy vegetable cluster	Starchy vegetable cluster vs. pasta/rice reference cluster	1.44 (0.72–2.90)	Sex, race, age, socioeconomic status
2015 Hashemi [30]	Iran	EWGSOP1	DXA	300 (54/246)	≥55	FFQ. Interviewer administered	Mediterranean dietary pattern	Highest tertile (T3) vs. lowest tertile (T1)	0.40 (0.17–0.97)	Age, sex, energy intake, physical activity, smoking, alcohol consume, drug consume, positive history of disease
2015 Kim [19]	Korea	Baumgartner’s criteria (aLM/ht^2^ is less than 7.23 kg/ht^2^ in men and 5.67 kg/ht^2^ in women)	DXA	1912 (1912/0)	≥65	FFQ. Interviewer administered	Vegetables and fruit	Highest quintile (T5) vs. lowest quintile (T1)	0.50 (0.21–1.24)	Age, education level, number of physician-diagnosed chronic conditions, BMI, smoking, alcohol drinking, physical activity, supplementary nutrient intake, age at menarche, oral contraceptive use, hormone use, quintiles of other food group consumptions
2016 Chan [31]	Hong Kong	AWGS2014	DXA	3957 (290/3667)	≥65	FFQ. Interviewer administered	Vegetables–fruit pattern score	Higher score vs. lower score	0.87 (0.73–1.04)	Age, BMI, energy intake, PASE, education level, smoking status, alcohol use, number of chronic diseases, GDS category, CSID category, living alone, marital status
2017 Mohseni [32]	Iran	EWGSOP1	BIA	250 (55/195)	≥45	FFQ. Interviewer administered	Mediterranean pattern	Highest tertile (T3) vs. lowest tertile (T1)	0.40 (0.17–0.89)	Age, physical activity, BMI, menopause duration, hypothyroidism, hormone replacement therapy, ACEi use, statin use, vitamin D use
2020 Koyanagi [15]	LMICs	SMM and either slow gait or low handgrip strength	None	14,585 (2290/12,295)	≥65	Two questions. Interviewer administered	Vegetable consumption	≥7/day vs. 0–1/day	0.99 (0.62–1.58)	Sex, age, education wealth, physical activity, smoking, alcohol consumption, BMI, number of chronic conditions, fruit consumption
2020 Li [33]	China	AWGS2014	BIA	861 (132/729)	≥65	FFQ. Interviewer administered	“Mushrooms-fruits-milk” pattern score	Q4 (0.49–6.12) vs. Q1 (−2.09–−0.66)	0.33 (0.14–0.77)	Age, gender, region, BMI, exercise activity, lifestyle, total dietary energy, smoke status, status of NCDs
2021 Fu [20]	China	EWGSOP2	BIA	591 (56/535)	≥40	FFQ. Interviewer administered	“Coarse cereals and vegetables” dietary patterns	Highest tertile (T3) vs. lowest tertile (T1)	0.37 (0.17–0.77)	Age, sex, smoking, physical activity
2021 Yokoyama [35]	Japan	AWGS2019	BIA	1606 (169/1437)	≥65	BDHQ. Self-administered	Dietary pattern 1 scores	Highest tertile (T3) vs. lowest tertile (T1)	0.57 (0.34–0.94)	Sex, age, study site, education, living alone, smoking habits, drinking habits, self-perceived chewing ability, frequency of going out, medical history, BMI, energy intake, MMSE
2022 Borges [36]	Spain	EWGSOP2	BIA	90 (27/63)	≥65	MEDAS. Interviewer administered	MEDAS categories	High (≥9) vs. low (1–8)	1.13 (0.37–3.96)	None
2022 Wang [38]	China	AWGS2014	BIA	2423 (391/2032)	≥60	FFQ. Self-administered	Vegetable pattern	Highest quartile (Q4) vs. lowest quartile (Q1)	0.54 (0.34–0.86)	Age, sex, BMI, physical activity, smoking status, drinking status, individual history of disease, total energy intake, depressive symptoms, household income, marital status, education level, employment status, the scores of other two dietary patterns
Cohort study										
2021 Yeung [34]	Hong Kong	AWGS2019	DXA	3992 (899/3093)	≥65	FFQ. Interviewer administered	Combined FV variety	T3 (≥38) vs. T1 (≤28)	0.94 (0.60–1.49)	Age, sex, BMI, current smoker, current drinker, live alone, education level, subjective social status, CSID category, number of chronic diseases, depressive symptoms, PASE score, daily energy intake, DQI-I score, daily amount of fruit and vegetable consumption
2022 Karlsson [37]	Sweden	EWGSOP2	DXA	257 (50/207)	Mean age 71	7-day diet record. Interviewer administered	Dietary pattern 2	Highest tertile vs. lowest tertile	0.40 (0.17–0.94)	Age, follow-up period, energy intake, education, physical activity, smoking, morbidity, BMI
2022 Park [24]	Korea	AWGS2014	DXA	801 (111/690)	70–84	24 h dietary recall. Interviewer administered	Vegetables	Highest quartile vs. lowest quartile	0.28 (0.13–0.59)	Age, sex, BMI, family type, marital status, education level, income level, smoking status, drinking status

Abbreviations: OR, odds ratio; CI, confidence interval; EWGSOP, European Working Group on Sarcopenia on Older People; DXA, dual-energy X-ray absorptiometry; FFQ, food frequency questionnaire; aLM, appendicular lean mass; BMI, body mass index; AWGS, Asian Working Group for Sarcopenia; PASE, Physical Activity Scale of the Elderly; GDS, Geriatric Depression Scale; CSID, Cognitive Screening Instrument for Dementia; BIA, bioelectric impedance analysis; ACEi, angiotensin converting enzyme inhibitor; SMM, low skeletal muscle mass; NCDs, non-communicable chronic diseases; BDHQ, validated brief self-administered diet history questionnaire; MMSE, Mini Mental State Examination Score; MEDAS, Mediterranean Diet Adherence Screener; FV, fruit and vegetable; DQI-I, Dietary Quality Index-International.

### 3.4. Result of the Meta-Analysis

**Figure 2:**
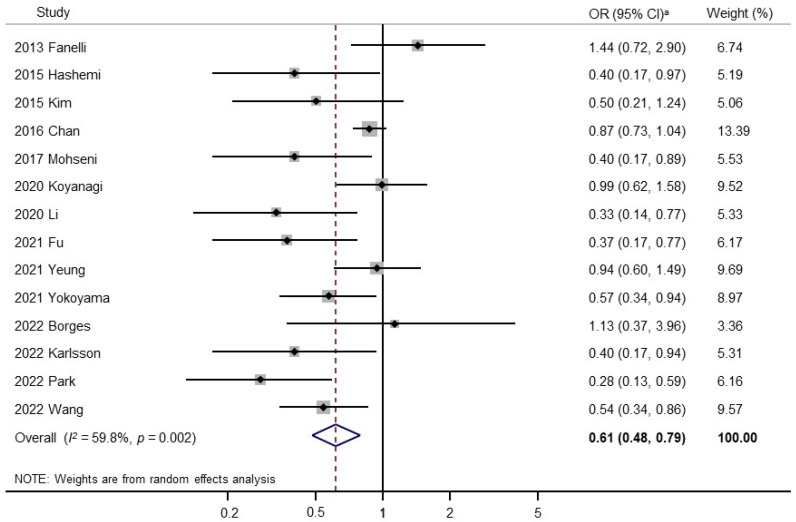
Forest plot of meta-analysis of studies of vegetable and fruit intake and sarcopenia. A random-effects model. ^a^ OR, odds ratio; CI, confidence interval [15,19,20,24,29,30,31,32,33,34,35,36,37,38].

**Figure 3:**
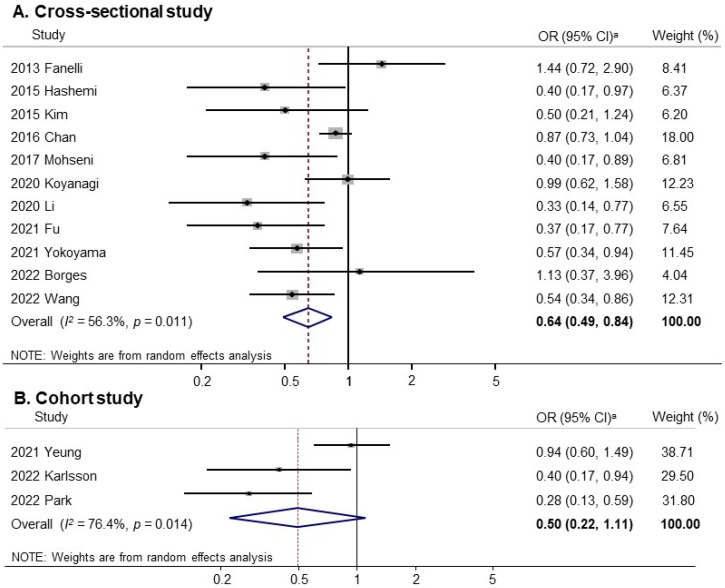
Forest plot of meta-analysis of studies of vegetable and fruit intake and sarcopenia by study design. A random-effects model. ^a^ OR, odds ratio; CI, confidence interval [15,19,20,24,29,30,31,32,33,34,35,36,37,38].

Figure 2 presents the association between vegetable and fruit consumption (highest versus lowest) and the risk of sarcopenia in a random-effects meta-analysis of all 14 observational studies. Overall, vegetable and fruit consumption was significantly associated with reduced risk of sarcopenia (OR, 0.61; 95% CI, 0.48 to 0.79; *I*^2^ = 59.8%). Figure 3 shows the results of the subgroup meta-analyses by study design. Vegetable and fruit consumption was significantly associated with reduced risk of sarcopenia in cross-sectional studies (OR, 0.64; 95% CI, 0.49 to 0.84; *I*^2^ = 56.3%; n = 11) but not in cohort studies (OR, 0.50; 95% CI, 0.22 to 1.11; *I*^2^ = 76.4%; n = 3). Publication bias was assessed using funnel plots and Egger’s test. The Begg’s funnel plot revealed an asymmetric result (Supplementary Figure S1). Therefore, there was evidence of publication bias in 14 studies (Egger’s test, *p* for bias = 0.03). A sensitivity analysis with the exclusion of one study by Chan et al. [31] led to the absence of publication bias (Egger’s test, *p* for bias = 0.14) (Supplementary Figure S2). The exclusion of this study from the pooled estimate did not affect the strength of the association (OR, 0.58; 95% CI, 0.44 to 0.76; *I*^2^ = 53.0%; n = 13) (Supplementary Figure S3). Therefore, the results must be interpreted with the overall significance of the association estimate.

### 3.5. Subgroup Meta-Analyses

Subgroup meta-analyses were performed by various factors such as sex, age, dietary patterns, definition criteria for sarcopenia, geographical region of the included participants, and methodological quality. Table 3 presents the results of the subgroup analyses. In subgroup analyses by sex, vegetable and fruit consumption was significantly associated with reduced risk of sarcopenia in men (OR, 0.60; 95% CI, 0.37 to 0.98; *I*^2^ = 63.9%; n = 4) but not in women (OR, 0.85; 95% CI, 0.62 to 1.17; *I*^2^ = 30.4%; n = 4). In subgroup analyses by age, vegetable and fruit consumption was also significantly associated with reduced risk of sarcopenia in age ≥60 (OR, 0.64; 95% CI, 0.49 to 0.83; *I*^2^ = 58.0%; n = 10). In subgroup analyses with dietary patterns, vegetable (OR, 0.66; 95% CI, 0.44 to 0.99; *I*^2^ = 62.7%; n = 5) and fruit (OR, 0.62; 95% CI, 0.42 to 0.91; *I*^2^ = 58.2%; n = 4) consumption, and a Mediterranean diet (OR, 0.68; 95% CI, 0.38 to 1.21; *I*^2^ = 65.2%; n = 4), significantly reduced the risk of sarcopenia. In subgroup analyses according to the definition criteria for sarcopenia, vegetable and fruit consumption was significantly associated with reduced risk of sarcopenia in groups with the definitions from AWGS 2014 (OR, 0.50; 95% CI≤, 0.29 to 0.87; *I*^2^ = 79.6%; n = 4) and EWGSOP2 (OR, 0.48; 95% CI, 0.27 to 0.88; *I*^2^ = 23.8%; n = 3) but not in groups with the definitions from AWGS 2019 (OR, 0.74; 95% CI, 0.45 to 1.21; *I*^2^ = 51.6%; n = 2) or EWGSOP1 (OR, 0.63; 95% CI, 0.26 to 1.53; *I*^2^ = 73.2%; n = 3).

## 4. Discussion

To the best of our knowledge, this is the first meta-analysis to focus on the association between sarcopenia and vegetable and fruit consumption in observational studies. We included studies diagnosing sarcopenia using measures of appendicular skeletal muscle mass, grip strength, and gait speed (or rising from a chair, etc.) strictly according to international diagnostic criteria. Subgroup meta-analyses were conducted based on age groups, study design, and other factors relevant to the risk of sarcopenia. Our findings suggest that vegetable and fruit intake significantly reduce the risk of sarcopenia. Subgroup analyses showed that vegetable and fruit consumption reduced sarcopenia risk in men and the age group of ≥60 years.

The effects of vegetable and fruit intake on muscle health have been reported [31,39]. However, there are limited meta-analysis studies regarding the association between vegetable and fruit consumption and sarcopenia. Besora-Moreno et al. [23] systematically reviewed and meta-analyzed 19 observational studies and nine randomized controlled trials (RCTs) to explore the association between antioxidant-rich foods, antioxidant supplements, and sarcopenia in adults aged ≥55 years. The meta-analysis results of RCTs showed that a high intake of fruit or vegetables, magnesium supplementation, and vitamin E plus vitamin D and protein significantly reduced the time to complete five stands. On the other hand, catechin supplementation was significantly associated with increased grip strength. In this study, we performed the following: (1) meta-analysis of only observational studies; (2) included only studies that assessed all sarcopenia parameters defined in the international standards for meta-analysis; (3) incorporated not only the quantity but also the patterns of fruit and vegetable consumption; and (4) included participants of a wide age range, including those aged ≥30 years, which distinguishes this study from previous research.

In this study, the consumption of vegetable and fruit proved to be a beneficial factor in reducing the risk of sarcopenia. The association between fruit and vegetable consumption and sarcopenia has been consistently reported [31,39]. Although the mechanism underlying the association between vegetable and fruit intake and sarcopenia is unclear, the antioxidant nutrients abundant in vegetables and fruits, as well as their compositional characteristics (alkalinity), are thought to have beneficial effects [40]. A possible mechanism for the association is that metabolic acidosis is closely associated with muscle wasting [41]; therefore, consuming alkaline foods such as vegetables and fruit may help to preserve muscle mass. Furthermore, phytochemicals abundant in vegetables and fruit can restore redox homeostasis in muscles and protect against damage induced by reactive oxygen species/reactive nitrogen species [18]. Moreover, vegetables and fruits are rich sources of vitamin C, which acts as an electron donor to reduce oxidative damage in muscles and decrease the concentration of inflammatory cytokines in the circulation [42,43]. Some studies suggest that vitamin C intake and serum vitamin C levels are significantly associated with skeletal muscle mass in middle-aged and older adults, and may be very useful in reducing age-related muscle loss [44].

This study found that vegetable and fruit consumption significantly reduced the risk of sarcopenia in men but not women. A study by Kim et al. [19], conducted in Korea, found a stronger association between fruit and vegetable consumption and sarcopenia in men than in women. In contrast, a study by Koyanagi et al. [15], conducted in six low- and middle-income countries, reported that the association between fruit consumption and sarcopenia was evident only in women but not in men. The differences in the association between fruit and vegetable consumption and sarcopenia by sex can be attributed to several factors. Different hormone profiles between men and women may lead to different causes of sarcopenia [45,46]. Sex hormones play an important role in maintaining skeletal muscle integrity. Testosterone acts as an anabolic factor that promotes muscle protein synthesis and regeneration, while estrogen has anti-inflammatory effects that protect skeletal muscles. However, these muscle-related functions of sex hormones may vary in their effect on muscle wasting with age or depending on the disease patterns [45].

The extent to which known sarcopenia risk factors, such as physical activity, affect individuals may vary by sex [47]. An earlier study reported that women may have more knowledge about the nutritional benefits of fruits and vegetables compared with men, which may lead to greater motivation to consume fruit and vegetables. This could lead to differences in the quantity of fruit and vegetables consumed between men and women and in the selection of nutritionally superior fruit and vegetables in women [48]. Future research should explore the effects of hormone secretion, lifestyle factors influencing muscle health, nutritional knowledge, and willingness to consume fruit and vegetables to investigate the relationship between fruit and vegetable consumption and muscle health.

The diagnostic criteria for sarcopenia vary widely. These criteria are defined differently according to race [49,50,51,52], and sarcopenia is diagnosed based on aspects of muscle mass, muscle function, and physical performance [49]. According to AWGS 2019, both the quantity and quality of muscle must be measured to diagnose sarcopenia [49]. Nevertheless, previous studies have reported associations with dietary intake using only some of the diagnostic criteria for sarcopenia [53,54]. There are no previous studies conducting meta-analyses of fruit and vegetable consumption and sarcopenia in observational studies. A systematic literature review of the association between antioxidant nutrient intake, including vegetables and fruit, and sarcopenia in observational studies [23] had limitations in separately examining muscle mass and strength. However, the meta-analysis in this study has strengths in including studies encompassing all three sarcopenia parameters (appendicular skeletal muscle mass, grip strength, and gait speed).

This meta-analysis has several limitations. First, there was a possibility of recall bias due to the cross-sectional design of some of the included studies. Cohort studies on the effects of vegetable and fruit consumption on sarcopenia are insufficient; therefore, only three cohort studies were included in this study. Second, we could not specify the variables for vegetable and fruit consumption due to insufficient data from previous studies on the association between vegetable and fruit consumption and sarcopenia.

Despite these limitations, this meta-analysis has several strengths. The main strength is that subgroup meta-analyses were performed in this study based on study design (cross-sectional and cohort), sex, age group, vegetable and fruit consumption patterns, diagnostic criteria for sarcopenia, and region. As there are no previous relevant systematic reviews and meta-analyses regarding the association between vegetable and fruit consumption and sarcopenia, this study will substantially contribute to the literature.

## 5. Conclusions

In conclusion, our meta-analysis showed that fruit and vegetable consumption significantly reduced the risk of sarcopenia, particularly in the age group of ≥60 years and in men. However, there is still insufficient systematic investigation or meta-analysis of the association between different variables related to vegetable and fruit consumption and sarcopenia. Therefore, as cohort studies provide a higher level of evidence than case–control studies, further prospective cohort studies should be conducted.

## Figures and Tables

**Table 2:** Methodological quality of the included studies based on the Newcastle-Ottawa Scale.

Cross-Sectional Study (n = 11)	Selection				Comparability	Exposure			Total
	Adequate Definition of Cases	Representativeness of Cases	Selection of Controls	Definition of Controls	Control for Important Factor or Additional Factor	Ascertainment of Exposure (Blinding)	Same Method of Ascertainment for Participants	Nonresponse Rate	
2013 Fanelli [29]	1	1	1	1	1	0	0	1	6
2015 Hashemi [30]	1	1	1	1	1	1	0	1	7
2015 Kim [19]	1	1	1	1	1	1	0	1	8
2016 Chan [31]	1	1	1	1	1	1	0	1	7
2017 Mohseni [32]	1	1	1	1	1	0	0	1	6
2020 Koyanagi [15]	1	1	1	1	1	0	0	1	6
2020 Li [33]	1	1	1	1	1	1	0	1	8
2021 Fu [20]	1	1	1	1	1	0	0	1	6
2021 Yokoyama [35]	1	1	1	1	1	1	0	1	8
2022 Borges [36]	1	1	1	1	0	0	0	1	5
2022 Wang [38]	1	1	1	1	1	1	0	1	7
Cohort Study (n = 3)	Selection				Comparability	Exposure			Total
	Representativeness of the Exposed Cohort	Selection of the Non-Exposed Cohort	Ascertainment of Exposure	Outcome of Interest was not Present at Start of Study	Control for Important Factor or Additional Factor	Assessment of Outcome	Follow-Up Long Enough for Outcomes to Occur	Adequacy of Follow-up of Cohorts	
2021 Yeung [34]	1	1	1	1	2	0	1	0	7
2022 Karlsson [37]	1	1	1	0	2	0	1	0	6
2022 Park [24]	1	1	1	0	1	0	0	1	5

Each study can receive up to one star for each item in the selection and exposure categories, and up to two stars in the comparability category.

**Table 3:** Subgroup meta-analyses of studies on vegetable and fruit intake and sarcopenia.

Factors	Number of Studies	Summary OR (95% CI)	Heterogeneity, *I*^2^ (%)
Study design			
Cross-sectional study	11	0.64 (0.49–0.84)	56.3
Cohort study	3	0.50 (0.22–1.11)	76.4
Sex			
Men	4	0.60 (0.37–0.98)	63.9
Women	4	0.85 (0.62–1.17)	30.4
Age			
65 years and older	9	0.65 (0.49–0.87)	58.4
60 years and older	10	0.64 (0.49–0.83)	58.0
Dietary patterns			
Vegetable	5	0.66 (0.44–0.99)	62.7
Fruit	4	0.62 (0.42–0.91)	58.2
Mediterranean diet	4	0.68 (0.38–1.21)	65.2
Definition of sarcopenia			
AWGS2014	4	0.50 (0.29–0.87)	79.6
AWGS2019	2	0.74 (0.45–1.21)	51.6
EWGSOP1	3	0.63 (0.26–1.53)	73.2
EWGSOP2	3	0.48 (0.27–0.88)	23.8
Region			
America	1	1.44 (0.72–2.90)	0.0
Asia	10	0.54 (0.40–0.72)	63.2
Europe	2	0.62 (0.23–1.69)	48.4
Methodological quality			
High quality	7	0.64 (0.48–0.84)	53.9
Low quality	7	0.60 (0.36–0.98)	67.4

Abbreviations: OR, odds ratio; CI, confidence interval; AWGS, Asian Working Group for Sarcopenia; EWGSOP, European Working Group on Sarcopenia on Older People.

## Supplementary Materials

The following supporting information can be downloaded at: https://www.mdpi.com/article/10.3390/nu16111707/s1, Table S1. PRISMA 2020 Checklist. Figure S1. Funnel plots for identifying publication bias in the meta-analysis of observational studies. Abbreviation: OR, Odd Ratio; SE, Standard Error. Figure S2. Funnel plots for identifying publication bias after exclusion study of Chan et al. 2016 [31]. Abbreviation: OR, Odd Ratio; SE, Standard Error. Figure S3. Forest plot of meta-analysis of studies on vegetable and fruit intake and sarcopenia after exclusion study of Chan et al. ^a^ Random-Effects Model. OR, Odd Ratio; CI, Confidence Interval.
