# Supplementary material for: Association of Dietary Vegetable and Fruit Consumption with Sarcopenia: A Systematic Review and Meta-Analysis
Source: Nutrients. 2024 May 30;16(11):1707. doi: 10.3390/nu16111707 (PMC11174889; doi:10.3390/nu16111707)
Supplement: Supplementary file 1 [file nutrients-16-01707-s001.zip › supplementaryfigures_수정.pdf]

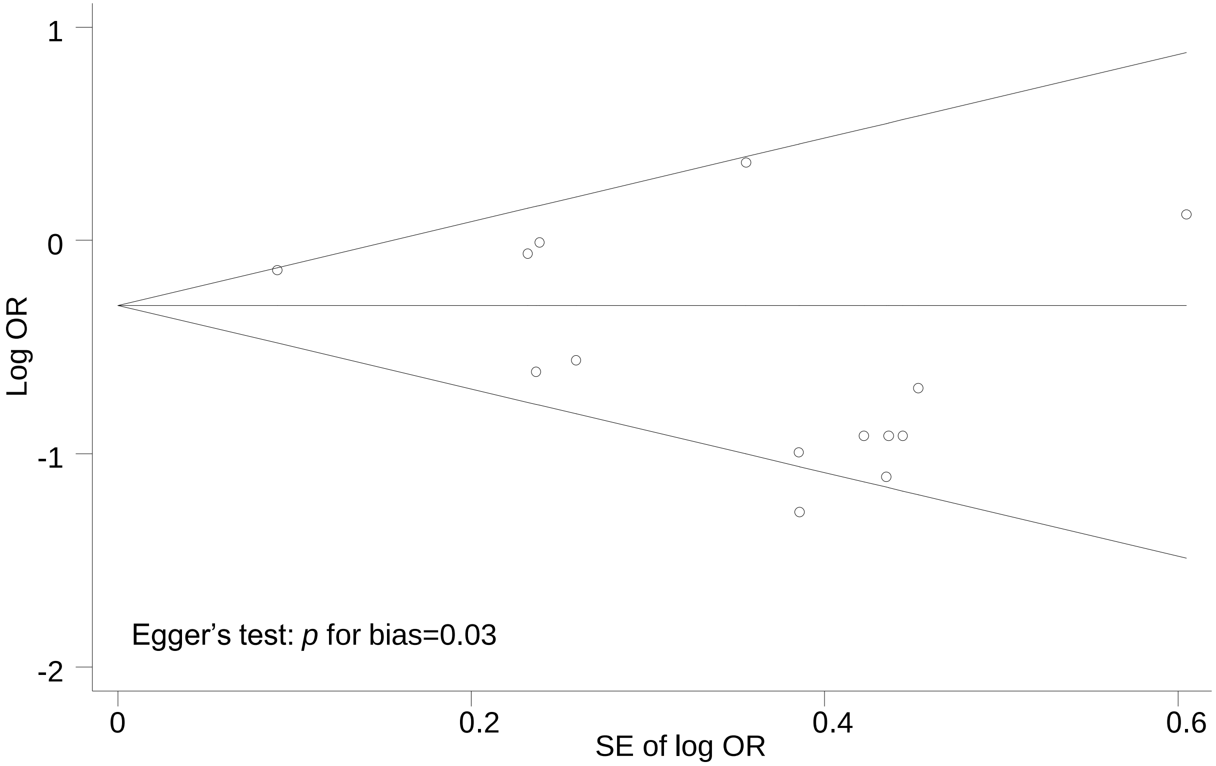

Supplementary Figure S1. Funnel plots for identifying publication bias in the meta-analysis of observational studies. Abbreviation: OR, Odd Ratio; SE, Standard Error.

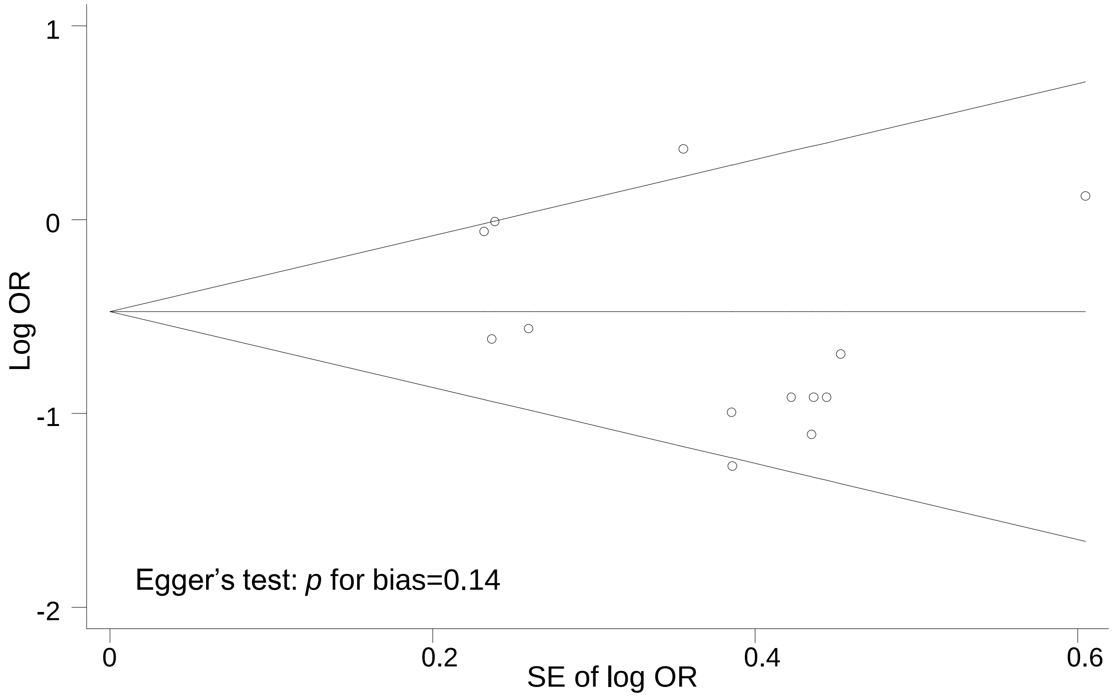

Supplementary Figure S2. Funnel plots for identifying publication bias after exclusion study of Chan et al. 2016 [31]. Abbreviation: OR, Odd Ratio; SE, Standard Error.

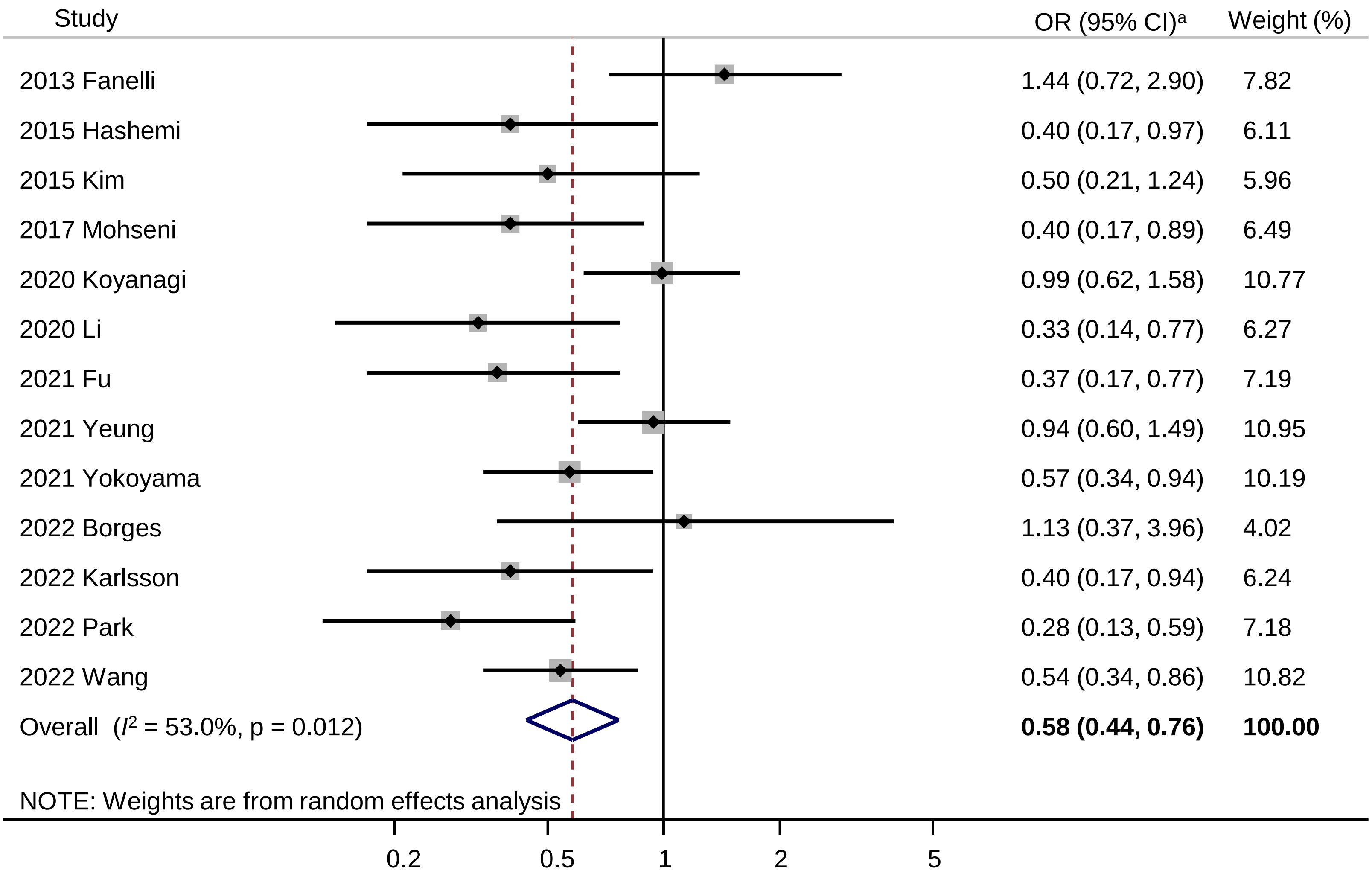

Supplementary Figure S3. Forest plot of meta-analysis of studies on vegetable and fruit intake and sarcopenia after exclusion study of Chan et al. 2016 [31]. [a] Random-Effects Model. OR, Odd Ratio; CI, Confidence Interval. [15,19,20,24,29–38].
